# Assessing the delivery error detection sensitivity of a new 2D detector prototype for carbon ion PSQA measurements

**DOI:** 10.1002/acm2.70580

**Published:** 2026-05-12

**Authors:** Javier Pérez‐Curbelo, Carlos M. Carvalho, Mansure Schafasand, Séverine Rossomme, Gabriela Llosá, Loïc Grevillot

**Affiliations:** ^1^ Instituto de Física Corpuscular (IFIC) CSIC‐UV Valencia Spain; ^2^ MedAustron Ion Therapy Center Wiener Neustadt Austria; ^3^ IBA Dosimetry Louvain‐la‐Neuve Belgium

**Keywords:** Carbon ion patient‐specific quality assurance, 2D ionization chamber array, MatriXX AiR, Delivery error detection, Detector sensitivity

## Abstract

**Background:**

Particle therapy requires stringent patient‐specific quality assurance (PSQA) to validate dose distributions delivered by the system.

**Purpose:**

This study presents preliminary results on the performance evaluation of the MatriXX AiR 2D ionization‐chamber array for patient‐specific QA (PSQA) in carbon‐ion therapy, comparing measured and planned dose distributions.

**Methods:**

A series of 10 test cases, including a treatment plan for a box‐shaped target (6‐cm side length), uniformly covered by the dose and nine patient‐specific treatment plans, were assessed. Measurements were conducted at two water equivalent depths using RW3 phantoms: beam entrance and within the Spread‐Out Bragg Peak (SOBP). The study examined the detector's ability to detect deviations by systematically modifying treatment plans, comparing the measured and planned dose utilizing gamma index analysis.

**Results:**

Passing rate analysis revealed depth‐dependent sensitivity to treatment plan delivery modifications. Measurements at the entrance depth showed better sensitivity to delivery changes than those taken within the SOBP

**Conclusions:**

The MatriXX AiR detector demonstrated strong potential for PSQA in carbon ion therapy. These findings support its application in clinical workflows to enhance treatment verification and accuracy.

## 1 INTRODUCTION

The complexity of particle therapy demands stringent patient‐specific quality assurance (PSQA) protocols to validate dose distributions and detect discrepancies between planned and delivered doses.^1^ PSQA in particle therapy typically involves verifying key treatment parameters, such as dose distribution, against the planned treatment. Detector arrays, such as MatriXX from IBA‐Dosimetry (Schwarzenbruck, Germany), have become a popular choice for PSQA in proton therapy due to their spatial resolution and efficiency in assessing dose distribution.^2^, ^3^, ^4^, ^5^, ^6^ Ionization chambers used in water phantoms (including three‐dimensional or motor‐driven designs) have traditionally been used worldwide for carbon ion therapy dosimetry but lacks dedicated medical software.^7^, ^8^, ^9^, ^10^ The new MatriXX AiR (IBA‐Dosimetry)—designed for ion beams delivered by pencil‐beam scanning, where individual spots create very high local dose rates^11^—offers a complete hardware‐and‐software solution that enables a more standardized PSQA approach similar to proton‐ and photon‐like methodologies. This study reports preliminary, single‐center results on the performance of the MatriXX AiR 2D array for PSQA in carbon‐ion therapy. The 2D detector was tested using a vertical pencil beam scanning carbon ion beam at EBG MedAustron in Wiener Neustadt.^12^, ^13^ The goal was to document a minimal, reproducible workflow and early performance indicators, rather than an exhaustive multicenter validation or device‐to‐device comparison. The detector's ability to flag controlled treatment plan perturbations—single energy‐layer deletions proximal or distal to the Spread‐Out Bragg Peak (SOBP) midpoint—within a standard clinical PSQA workflow was assessed.

## 2 MATERIALS AND METHODS

The MatriXX AiR (IBA Dosimetry, Schwarzenbruck, Germany) is a two‐dimensional air‐vented parallel‐plate ionization chamber array developed for quality assurance in particle therapy. The detector is equipped with an array of 1521 air‐vented ionization chambers, arranged in a 39 × 39 grid with a center‐to‐center spacing of 6.5 mm, covering an active measurement area of 25 cm × 25 cm. Each chamber has a diameter of 3.2 mm and an electrode gap of 1 mm, corresponding to a sensitive volume of approximately 8 ${\mathrm{mm}}^{3}$. In comparison, traditional carbon ion patient QA at EBG MedAustron is based on the 3D‐block system (PTW, Freiburg) features 24 PinPoint ionization chambers type 31015 (PTW, Freiburg) with a volume of 0.03 ${\mathrm{cm}}^{3}$ and a spacing of 10–12 mm.^14^

Earlier MatriXX systems designed primarily for photon IMRT/VMAT verification consisted of 1020 chambers arranged in a 32 × 32 grid with 7.62‐mm spacing and substantially larger chamber volumes (0.08 ${\mathrm{cm}}^{3}$).^2^, ^3^, ^4^ The MatriXX AiR differs in chamber geometry, most notably through its reduced 1‐mm electrode gap and smaller sensitive volume. This modification is intended to minimize ion recombination and to allow accurate measurements at high instantaneous dose rates, as encountered in pencil beam scanning particle therapy.

Given the differences in detector geometry and the distinct beam delivery characteristics of particle therapy—such as spot‐wise irradiation, layer‐by‐layer energy modulation, and steep lateral and depth dose gradients—detector performance cannot be directly extrapolated from previous validations of photon‐based MatriXX systems. Dedicated characterization under clinical particle beam conditions is therefore required prior to implementation in patient‐specific QA.

**FIGURE 1:**
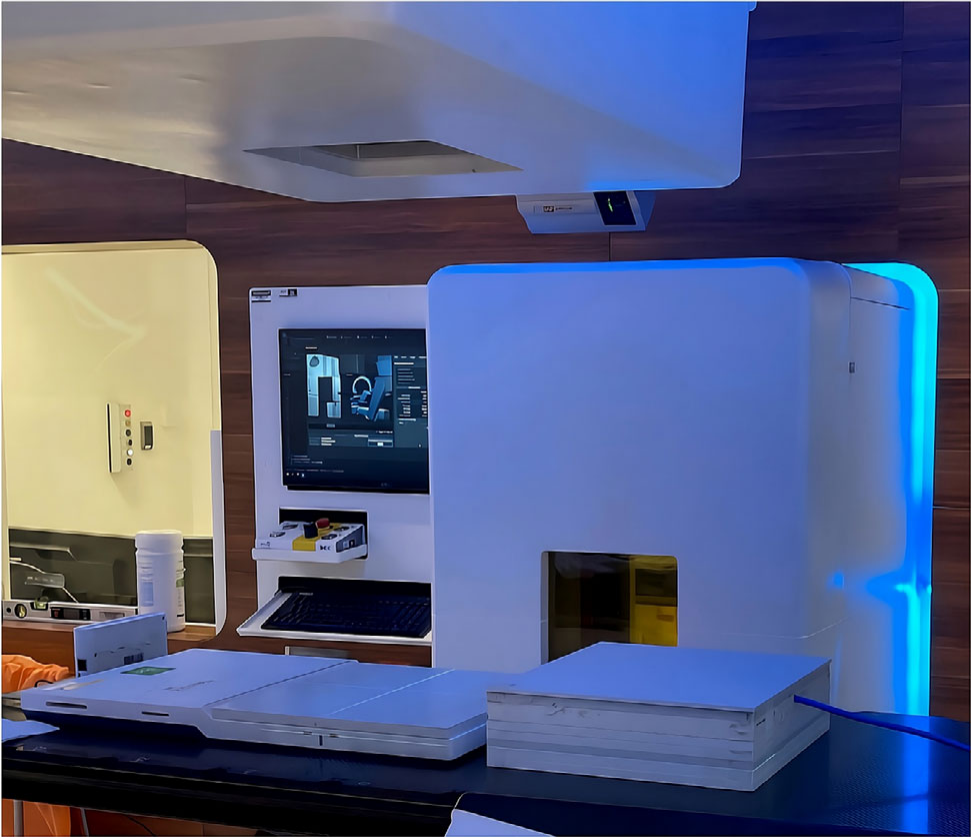
Experimental setup for MatriXX AiR cross‐calibration using a ROOS ionization chamber.

Before proceeding with dose measurements with the MatriXX AiR, a cross‐calibration was performed using a ROOS ionization chamber (PTW Freiburg, Germany) to determine the dose conversion factor that has to be applied to the response of the MatriXX AiR to convert counts to Gray. For this cross‐calibration, the MatriXX AiR response was taken from the central ionization chamber (central electrode) at isocenter. Relative calibration factors for the full detector array were applied using the vendor 2D calibration file provided for the MatriXX AiR, which defines the response of each chamber relative to the central chamber. The ROOS chamber was cross‐calibrated against a Farmer type ionization chamber and is used as reference dosimeter for reference dosimetry at MedAustron. Both detectors were placed at the isocenter and measurements were performed at 2‐cm water‐equivalent depth (WED). Figure 1 illustrates the setup used for the MatriXX AiR during cross‐calibration. The calibration was performed using a 346.6‐MeV/u carbon ion beam and validated by comparing MatriXX measurements against the ROOS chamber in three 3D box treatment plans at measurement depths ranging from 6 cm up to 25‐cm WED.

To evaluate the performance of the 2D detector array in detecting delivery errors, measurements on a diverse set of nine patient‐specific treatment plans and a box‐shaped target (6‐cm side length) were conducted. These treatment plans were selected to cover a range of clinical scenarios, including variations in the number of energy layers, planning target volume (PTV) sizes, and target depths. Across the investigated patient cases, the field sizes (as defined in the TPS at isocenter) ranged from 37 × 55 ${\mathrm{mm}}^{2}$ (Patient 2) up to 188 × 171 ${\mathrm{mm}}^{2}$. For all cases, the MatriXX AiR was at isocenter, aligned with in‐room lasers, with 1 cm of RW3 plate on top of the active area, enabling a straightforward and rapid setup. This setup corresponds to a WED estimated to be about 2 cm, referred to as the entrance depth throughout this work. To study the effectiveness of the 2D array to detect discrepancy between treatment plans and delivered doses, three specific cases were selected for in depth investigations: two from the nine patient‐specific treatment plans (“Patient 1” and “Patient 5”) and Box 6. For these cases, the original treatment plans (“nominal plan”) were modified by removing one energy layer with a nominal range shallower than the SOBP midpoint, hereafter the “proximal‐layer‐omission plan,” and one energy layer with a nominal range deeper than the SOBP midpoint, hereafter the “distal‐layer‐omission plan.” Measurements were repeated for these modified treatment plans, both at the middle of the SOBP and entrance depth. The “nominal plan” measurements served as a baseline for comparison against measurements using “nominal,” “proximal‐layer‐omission” and “distal‐layer‐omission” plans.

Dose distributions for the QA plans were calculated in the RayStation v.2024A SP1 (RaySearch Laboratories, Sweden) treatment planning system (TPS) in an RW3 phantom using the Pencil Beam v2024A dose calculation algorithm, and imported into the myQA software (IBA‐Dosimetry, Schwarzenbruck, Germany) for comparison with measurements. The comparison was based on global gamma analysis, with a dose criteria 2 or 3% and a distance‐to‐agreement (DTA) criterion of 2 mm, and a 10% low‐dose threshold (MatriXX measurements was used as reference, following the recommendations from IBA‐Dosimetry). In addition, doses at the central point were extracted.

## 3 RESULTS

**FIGURE 2:**
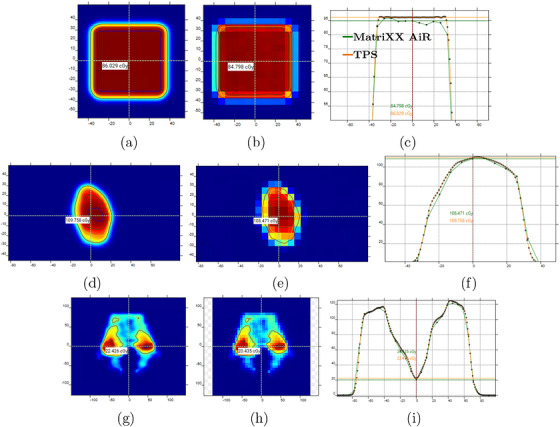
(a) and (b), (d) and (e), (g) and (h) show the nominal dose maps at the entrance depth for Box 6, Patient 2, and Patient 5, obtained from the TPS and MatriXX AiR, respectively. Panels (c), (f), and (i) present the corresponding lateral dose profiles, comparing the MatriXX AiR measurements (green) with the TPS reference plane (orange).

During the assessment of the cross‐calibration process, all 3D plans showed consistent agreement across geometries. Deviations of 0.7, 1.3, and 1.5% were obtained for 3D box treatment plans of 6‐, 8‐, and 10‐cm side lengths measured at depths of 6‐, 15‐, and 25‐cm WED, respectively, when comparing MatriXX AiR and ROOS chamber measurements. Based on this calibration, the results obtained for the test cases measured at the entrance depth are summarized in Table 1. Under the less stringent criteria (3%/2 mm), all test cases achieved passing rates above 99%. When the 2%/2‐mm criteria were applied the passing rate was lower, especially for Box 6, with 82.4%. Figure 2 presents the TPS and MatriXX AiR dose planes at the entrance depth together with lateral dose profiles for the Patient 2 (smallest‐field case), Patient 5 (large field and high central axis dose error) and the Box 6 cases. Those profiles provide additional qualitative context for the gamma passing rates reported in Table 1.

**TABLE 1:** Summary of gamma index results for all patient cases, using both gamma index criteria, 3%/2 mm and 2%/2 mm.

	3% and 2 mm	2% and 2 mm	Relative dose error (%)	PTV (${\mathrm{cm}}^{3}$)	Field size (${\mathrm{mm}}^{2}$)	Energy range (MeV/u)	No. of layers
Test cases	passing rate (%)	passing rate (%)	central point				
Box 6	99.4	82.4	1.5	216.0	73 × 73	120.0 ‐ 219.9	34
Patient 1	100.0	99.1	2.5	1723.5	188 × 171	120.0–328.7	83
Patient 2	100.0	96.4	1.2	45.9	37 × 55	157.5–237.4	30
Patient 3	98.5	96.5	−10.3	606.1	115 × 104	146.1–260.0	43
Patient 4	99.6	98.5	−0.3	312.5	162 × 100	135.9–248.3	41
Patient 5	100.0	100.0	9.7	847.9	165 × 166	171.4–362.0	71
Patient 6	100.0	99.8	−0.3	1307.6	188 × 170	141.1–298.8	62
Patient 7	100.0	97.9	1.6	2047.6	185 × 171	135.9–293.4	61
Patient 8	100.0	99.0	2.0	2061.9	188 × 169	134.2–385.5	105
Patient 9	100.0	100.0	−1.8	299.69	155 × 156	135.9–250.6	42

**FIGURE 3:**
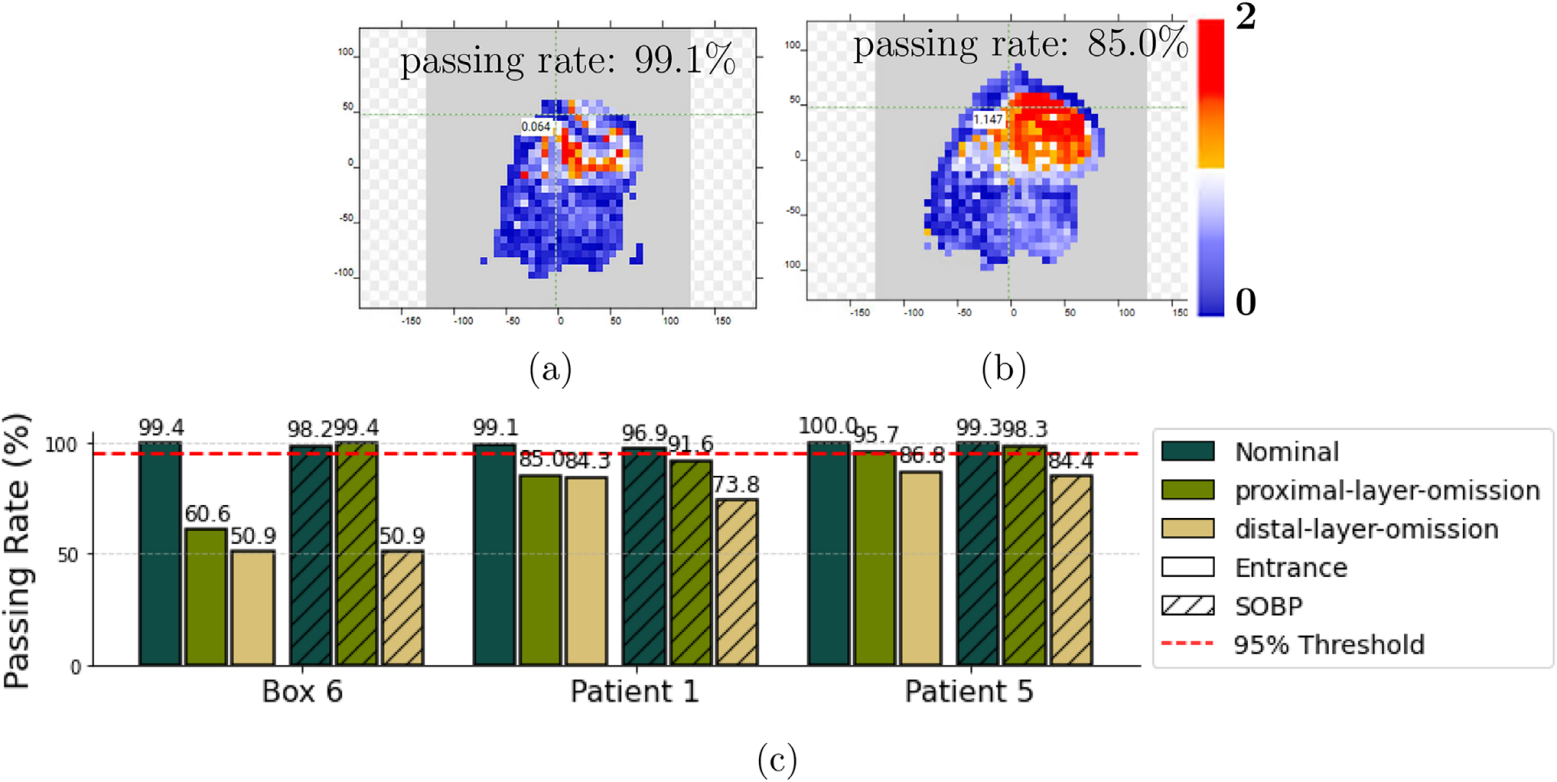
(a) and (b) Gamma index map for Patient 1 at the entrance depth and the “nominal” and “proximal‐layer‐omission” plans, respectively. (c) Passing rates for “nominal” and modified treatment plans at the entrance and the middle of the SOBP.

For these plans, an energy layer spacing of 2 mm was used. Removing one layer meant a jump of 4 mm instead of 2 mm in range. Figures 3a and 3b present an exemplary gamma index map for Patient 1 and Figure 3c shows the gamma index passing rate for the three specific cases, comparing the “nominal plan,” “proximal‐layer‐omission plan,” and “distal‐layer‐omission plan” for the two depths (entrance and middle of the SOBP). These results were obtained using a 3%/2‐mm criterion for the Box 6 and a 2%/2‐mm criterion for “Patient 1” and “Patient 5.”

## 4 DISCUSSION

As shown in Table 1, for the less stringent 3%/2‐mm criterion, all test cases achieved passing rates above 99%, indicating that this condition has limited sensitivity in distinguishing variations in dose agreement. When applying the stricter 2%/2‐mm criterion, all patient cases maintained passing rates above 95%, except for Box 6. The decrease in passing rate observed for Box 6 under the 2%/2‐mm criterion is primarily driven by dose differences rather than spatial agreement, as evidenced by the recovery of the passing rate to 99.4% when a 3%/2‐mm criterion is applied.

With respect to patient cases, the result do not show clear trends as a function of the field size, suggesting the MatriXX AiR is well suited for PSQA from small field sizes up to large field sizes as evaluated in this study. The two outliers in dose at the central electrode (Patients 3 and 5) in Table 1 corresponding to low doses and large gradient, where the usage of the DTA from the gamma analysis method becomes relevant. Gamma analysis is a powerful tool, but many details in the settings and clinical analysis must be considered for PSQA (beyond the scope of this publication) and we refer the users to the AAPM TG 219^15^ for more details.

Because the clinical vertical beam line limits the maximum deliverable field size to 188 × 171 ${\mathrm{mm}}^{2}$, fields near the maximum measurable area of the MatriXX AiR were not assessed. Overall, within the field‐size range represented in this study (approximately 37 × 55 ${\mathrm{mm}}^{2}$ up to 188 × 171 ${\mathrm{mm}}^{2}$), the MatriXX AiR showed consistently high gamma passing rates across patient plans, suggesting stable performance over the clinically relevant field sizes available in our cohort. This supports the applicability of the MatriXX AiR for PSQA in carbon‐ion treatments delivered with comparable field dimensions under the measurement conditions investigated here.

According to the results, the 2%/2‐mm criteria and 95% threshold, for the passing rate seem to be adequate candidate for evaluating dose distribution agreement with the MatriXX AiR. This threshold is consistent with commonly used standards for PSQA in ion therapy. For instance, Arjomandy et al.^2^ and Zhu et al.^3^ applied a 3%/3‐mm gamma index with a passing rate threshold of 90% to assess the accuracy of proton therapy treatment plans using the MatriXX detector. In contrast, Mackin et al.^4^ used more stringent criteria 2%/2 mm for gamma index analysis, with passing rates typically above 85%.

Regarding the MatriXX's efficiency in detecting errors, the passing rates in Figure 3c reveal clear differences between the “nominal plan,” “proximal‐layer‐omission plan,” and “distal‐layer‐omission plan” at both entrance and the middle of the SOBP. At the entrance, the MatriXX detector is sensitive to treatment plan modifications for both the “proximal‐layer‐omission plan” and “distal‐layer‐omission plan” scenarios. In contrast, at the SOBP, a smaller drop in the passing rate is observed for “proximal‐layer‐omission plan.” However, the change may be too small to reliably conclude that the missing energy layer was detected. For “distal‐layer‐omission plan,” the removed energy layer does impact the dose distribution at the SOBP, resulting in a more pronounced drop in the passing rate. These findings emphasize the importance of measurement depth in detecting treatment plan discrepancies. For the layer‐omission scenarios evaluated in this work, entrance depth measurements showed higher sensitivity for identifying such errors. Further investigations with a larger cohort of patients under varying clinical conditions, for example, including cases with a range shifter and missing, displaced, overdosed, or underdosed spots, even smaller field sizes and larger field sizes, considering, for example, patched fields treatments are warranted to confirm these findings and explore the detector's performance in more complex scenarios.

## 5 CONCLUSIONS

The MatriXX AiR detector demonstrated robust performance in identifying dose discrepancies in carbon ion therapy, particularly when analyzed using the stricter 2%/2‐mm gamma index criterion. In the context of the intentional layer‐omission test used in this preliminary evaluation, measurements performed at the beam entrance depth were more sensitive overall to delivery errors than those conducted within the SOBP, but in particular for proximal‐layer omission. The overall results confirm the detector's potential for clinical implementation in carbon ion dosimetry and PSQA.

## AUTHOR CONTRIBUTIONS

**Javier Pérez‐Curbelo**: Methodology; investigation; writing—review and editing. **Carlos M. Carvalho**: Methodology; writing—review and editing. **Mansure Schafasand**: Software; writing—review and editing. **Séverine Rossomme**: Methodology; writing—review and editing. **Gabriela Llosá**: Supervision; funding acquisition; writing—review and editing. **Loïc Grevillot**: Supervision; project managment; methodology; writing—review and editing.

## CONFLICT OF INTEREST STATEMENT

IBA Dosimetry (Schwarzenbruck, Germany) supported this work by providing access to MatriXX AiR prototypes and covering the open‐source publication fees.
